# Altitude and hillside orientation shapes the population structure of the *Leishmania* infantum vector *Phlebotomus ariasi*

**DOI:** 10.1038/s41598-020-71319-w

**Published:** 2020-09-02

**Authors:** Jorian Prudhomme, Thierry De Meeûs, Céline Toty, Cécile Cassan, Nil Rahola, Baptiste Vergnes, Remi Charrel, Bulent Alten, Denis Sereno, Anne-Laure Bañuls

**Affiliations:** 1 MIVEGEC Univ Montpellier, IRD, CNRS, Centre IRD, 911 avenue Agropolis, 34394 Montpellier, France; 2 INTERTRYP, IRD, Cirad, Univ Montpellier, Montpellier, France; 3 Unité des Virus Emergents (UVE: Aix Marseille Univ, IRD 190, INSERM 1207, IHU Méditerranée Infection), 13385 Marseille, France; 4 ESRL Laboratories, Department of Biology, Ecology Section, Faculty of Science, Hacettepe University, 0680 Beytepe, Ankara Turkey

**Keywords:** Genetic variation, Entomology, Ecological genetics

## Abstract

Despite their role in *Leishmania* transmission, little is known about the organization of sand fly populations in their environment. Here, we used 11 previously described microsatellite markers to investigate the population genetic structure of *Phlebotomus ariasi*, the main vector of *Leishmania infantum* in the region of Montpellier (South of France). From May to October 2011, we captured 1,253 *Ph. ariasi* specimens using sticky traps in 17 sites in the North of Montpellier along a 14-km transect, and recorded the relevant environmental data (e.g., altitude and hillside). Among the selected microsatellite markers, we removed five loci because of stutter artifacts, absence of polymorphism, or non-neutral evolution. Multiple regression analyses showed the influence of altitude and hillside (51% and 15%, respectively), and the absence of influence of geographic distance on the genetic data. The observed significant isolation by elevation suggested a population structure of *Ph. ariasi* organized in altitudinal ecotypes with substantial rates of migration and positive assortative mating. This organization has implications on sand fly ecology and pathogen transmission. Indeed, this structure might favor the global temporal and spatial stability of sand fly populations and the spread and increase of *L. infantum* cases in France. Our results highlight the necessity to consider sand fly populations at small scales to study their ecology and their impact on pathogens they transmit.

## Introduction

*Phlebotomus* sand flies are vectors of medically important pathogens, such as *Leishmania*, the causative agent of leishmaniasis^1^, and arthropod-borne viruses (Toscana virus, Naples virus, and Sicilian virus)^2^. *Phlebotomus ariasi* Tonnoir, 1921 is the predominant sand fly species in the French Cevennes region^3^, and one of the two proven vectors, with *Phlebotomus perniciosus* Newstead, 1911, of leishmaniasis, which is caused by *Leishmania infantum* in the South of France^4^. Sand flies are abundant in suburban and rural environments and are often close to human and domestic animal populations^4,5^. *Phlebotomus ariasi* is found resting in houses, animal sheds, caves and weep holes in walls, near roads, and in villages. This species has a wide geographic distribution, including many countries of the Western Mediterranean region, such as Algeria, France, Italy, Morocco, Portugal, Spain, and Tunisia^6–16^.

During the last 10 years, the risk of emergence or re-emergence of leishmaniasis^17^ and phlebovirus infections^18^ has increased in France, probably linked to the recent extension of the vector distribution. However, the biology and ecology of *Ph. ariasi*, one of the main vectors of *L. infantum*, remain poorly known.

Only few studies have been performed on this species. Analysis of cuticular hydrocarbons highlighted the presence of two *Ph. ariasi* populations (sylvatic and domestic) in the Cevennes region^19^. Studies based on isoenzyme data^20,21^, random amplified polymorphic DNA^22^, and mitochondrial cytochrome b (*cyt*b) gene sequences^11^ showed differences among *Ph. ariasi* populations in known leishmaniasis foci in Europe. Finally, two microsatellite loci and other genetic markers were used to understand *Ph. ariasi* expansion in Europe during the Pleistocene glacial cycles^23^.

However, no population genetics study tackled the influence of geographic (spatial organization) together with environmental (altitude and hillside) factors in the distribution of sand flies. Therefore, the aims of this work were to study the structure of *Ph. ariasi* populations at a local scale, and the impact of environmental factors (geographical distance, altitude, and hillside) on their spatial organization. For this purpose, sampling was performed in a well-documented area (Roquedur, Hérault, France), in which sand fly populations were already ecologically^24^ and morphometrically^25^ described, and where human and canine leishmaniasis caused by *L. infantum* are endemic^5,26^. The analysis was carried out using 11 previously described microsatellite loci for *Ph. ariasi*^27^.

## Results

### Genotyping

In total, 1,253 sand flies were genotyped using the 11 loci described in the Supplementary Table S1. Sand fly DNA samples in which more than six loci could not be amplified were removed from the analyses (n = 54 individuals, Group “/” in Supplementary Table S1).

### Bayesian clustering

**Figure 1:**
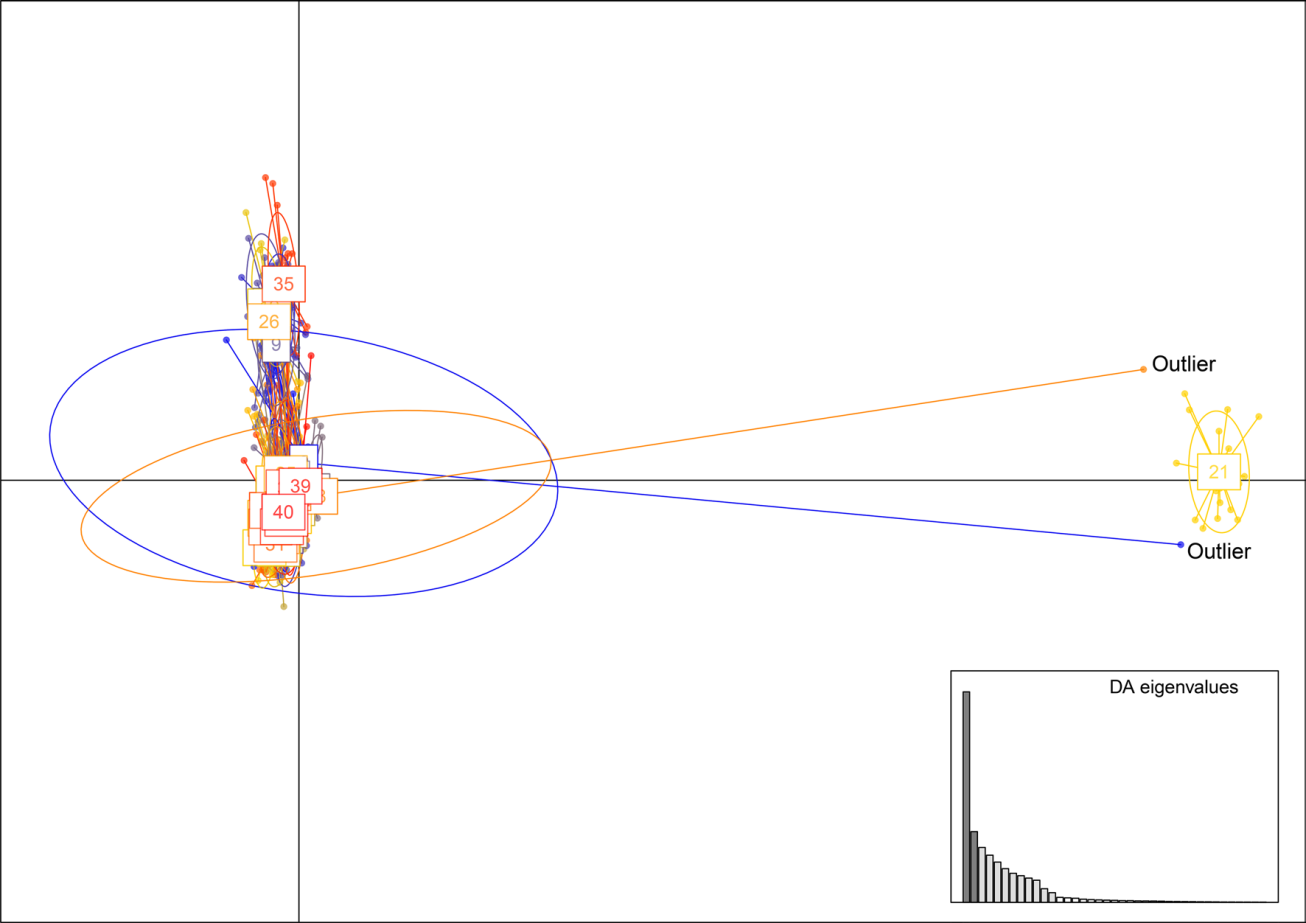
Genetic variation among the captured *Phlebotomus ariasi* individuals. Scatter plot of individuals based on the first two axes (created from the optimum 13 principal components) of the DAPC. The inset shows the amount of variation represented by the discriminant analysis eigenvalues. Points and ellipses are colored according to the groups defined by the DAPC. Misassigned individuals (outliers) are indicated.

Discriminant analysis of principal components (DAPC) identified 40 clusters with a mean assignment probability *P*_Ass_ = 0.8568. Twenty individuals were grouped in one strongly differentiated cluster (cluster 21 with *P*_Ass_ = 1) (Fig. 1). Moreover, two outliers (one from cluster 2 and one from cluster 28) were close to cluster 21. Bayesian Analysis of Population Structure (BAPS) found 28 clusters (probabilities for number of clusters = 0.93641). A cluster of 22 individuals (BAPS cluster 28) included the 20 individuals from DAPC cluster 21 and the two outliers highlighted by DAPC. The optimal number of clusters found by STRUCTURE HARVESTER (used to visualize the results of STRUCTURE analysis) was two with ΔK = 16.03 (the second biggest ΔK was 2.55). Surprisingly, the clusters found by STRUCTURE did not match the DAPC or BAPS results. These two clusters grouped individuals from several stations with no obvious relation with any ecological or geographical parameter, and with a very small average assignment probability of individuals to their cluster (*P*_Ass_ = 0.53683).The partition found by STRUCTURE and STRUCTURE harvester with K = 2 is probably meaningless.

**Figure 2:**
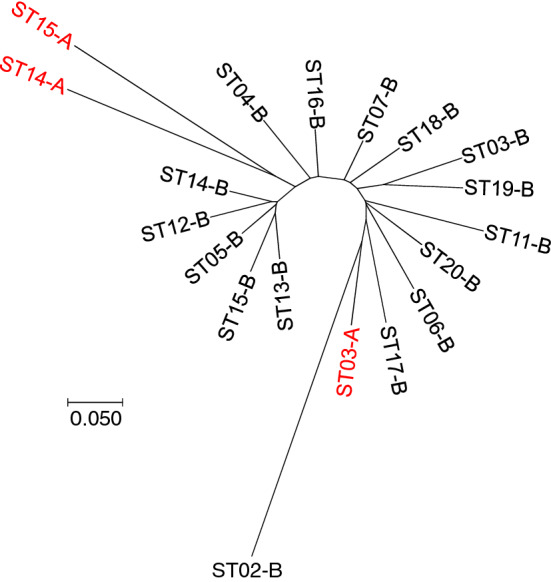
Dendrogram (NJTree) of the genetic relationship between subsamples defined as combinations of group A or B and sampling station (ST) based on Cavalli-Sforza and Edwards chord distance calculated with six loci (*Aria2*, *Aria3*, *Aria4*, *Aria5*, *Aria13,* and *Aria14*). Outlier individuals (Group A) are indicated in red. The same tree was obtained also using 10 loci (excluding *Aria1*).

*Cyt*b fragment sequencing of seven individuals from cluster 21 (# 12, 17, 18, 19, 856, 872, and 874) and one of the two outliers (# 23, cluster 2) showed 99–100% similarity with *Ph. ariasi* (GenBank accession number: KP685539.1, and sequences in Supplementary File 1). Therefore, the taxonomic status of the 22 outliers could not be elucidated. These 22 individuals (Group A in Supplementary Table S1) were all males captured in three stations (ST02, ST11 and ST12). Neither particular environmental condition nor specific morphological character was recorded for these individuals compared with the other sand fly specimens. The 22 outliers were homozygous for allele 204 at the *Aria1* locus. This allele was not found in the other 1,177 individuals (Group B in Supplementary Table S1). The dendrogram (NJTree) seems to exclude two subsamples A from all other subsamples, while a third one appeared fully included within group B (Fig. 2). This structuring appears to be robust since the same tree was obtained with 6 (*Aria2*, *Aria3*, *Aria4*, *Aria5*, *Aria13*, and *Aria14*, loci selected after Linkage Disequilibrium (LD) and F-statistics analyses, see below) or 10 loci (excluding *Aria1*).

Except when specified otherwise, the 22 individuals of Group A were removed from the analyses to prevent Wahlund effects.

### Locus selection for the analyses of Group B sand flies

The *Aria1* locus was almost monomorphic (*H*_S_ = 0.06) and was removed from the data. Using regression approach we detected a marginally not significant Short Allele Dominance (SAD) for the *Aria14* locus (*p value* = 0.0507). As SAD results from a preferential amplification of the shortest allele in heterozygous individuals^28^, the *Aria14* microsatellite profile of each homozygous individual was checked again following recommendation suggested in De Meeûs et al.^29^, corrected, and then analyzed using GENEMAPPER 4.0. After correction, SAD could not be detected on this locus any longer (*p* *value* = 0.0731).

The Micro-Checker analysis suggested stutter artifacts at five loci (*Aria2*, *Aria3*, *Aria10*, *Aria14*, and *Aria15*). The unilateral exact binomial test indicated that the proportion of significant stuttering was not significantly higher than the expected proportion under the null hypothesis for *Aria2* (*p value* = 0.2078), *Aria3* (*p value* = 0.2078) and *Aria14* (*p value* = 0.5819). Nevertheless, it was marginally not significant for *Aria10* (*p value* = 0.0503) and highly significant for *Aria15* (*p value* = 9.728e−06). Therefore, alleles close in size were pooled, avoiding pooling together only rare alleles (i.e., presence of at least one frequent allele in the pool) for these two loci as recommended^29^. As the *Aria10* locus became monomorphic after pooling, this locus was removed from the analysis. For *Aria15*, alleles 117, 119 and 121 were pooled with allele 123; allele 127 was pooled with allele 125; and allele 131 was pooled with allele 129. However, after correction, the Micro-Checker analysis and the unilateral exact binomial test (*p value* = 9.728e−06) highlighted that stuttering still affected this locus. Therefore, *Aria 15* was also removed from the analysis.

**Figure 3:**
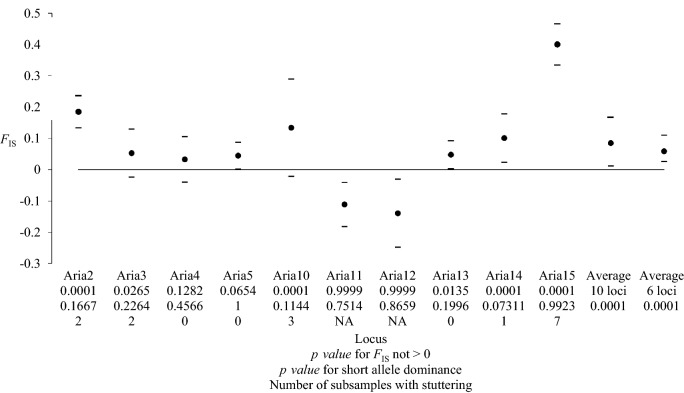
Deviation from the genotypic proportions expected for panmixia as measured by *F*_IS_ in *Phlebotomus ariasi* for each microsatellite locus and averaged across loci. For each locus, the 95% CI values for subsamples obtained with the jackknife over subsamples is represented with dashes. The 95% CI values for the average, with 10 and 6 loci, were obtained by 5,000 bootstraps over loci. The results of tests for panmixia, short allele dominance, and number of subsamples with stuttering for each locus are also indicated.

**Figure 4:**
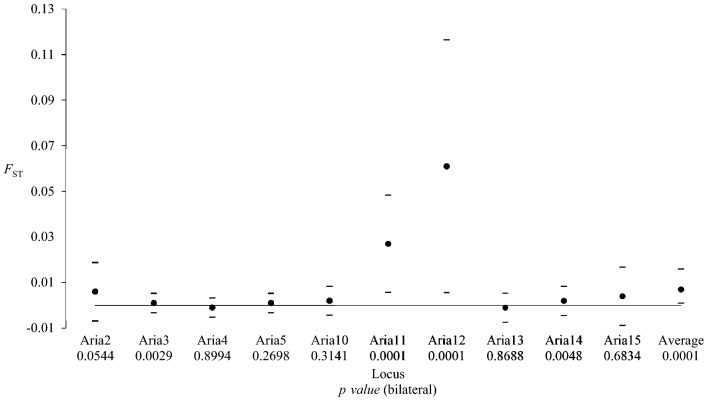
Effect of subdivision (*F*_ST_ value) in *Phlebotomus ariasi* for each microsatellite locus and averaged across loci. For each locus, the 95% CI value calculated with the jackknife over subsamples is represented with dashes. The 95% CI of average was obtained by 5,000 bootstraps over loci.

On the remaining 9 loci, the linkage disequilibrium (LD) analysis indicated that 7 locus pairs (out of 11) displayed a significant LD (19.4%). Three of these locus pairs (*Aria11* and *Aria 12*, *Aria 11* and *Aria 13*, *Aria 12* and *Aria14*) remained significant after Benjamini and Yekutieli adjustment. *Aria11* and *Aria12* displayed outlier profiles with strongly negative *F*_IS_ (Fig. 3), above average *F*_ST_ (Fig. 4), large *F*_IS_ and *F*_ST_ variance (Figs. 3, 4), and strong LD. These results suggested the non-neutrality of these loci. Therefore, *Aria11* and *Aria12* were also removed from the analysis.

To check the result stability, additional DAPC analyses were performed by successively removing the following loci *Aria1*, *Aria10*, *Aria11*, *Aria12*, and *Aria15*. As long as *Aria1* was kept, the obtained pattern was the same as in Fig. 2 (data not shown). With the remaining six loci (*Aria2*, *Aria3*, *Aria4*, *Aria5*, *Aria13*, and *Aria14*), DAPC and BAPS provided no clear structure. This suggested that the distinction between group A (the 22 outliers) and group B (the 1,177 remaining individuals) only relied on the *Aria1* locus (Supplementary Table S1).

### Linkage disequilibrium and *F-*statistics with the six remaining loci

With the remaining six loci (*Aria2*, *Aria3*, *Aria4*, *Aria5*, *Aria13,* and *Aria14*), the proportion of significant LD tests was 13.3% (two locus pairs), but none of these tests remained significant after Benjamini and Yekutieli adjustment. There was still a rather small but significant heterozygote deficit in subsamples (average *F*_IS_ = 0.059, *p value* = 0.0001). Variation across loci appeared pronounced (Fig. 3), but the relationship between *F*_IS_ and *F*_ST_ was not significant (Spearman's *ρ* = 0.2319, *p value* = 0.3292). There was no relationship between the number of missing data at each locus and the *F*_IS_ values (*ρ* = − 0.029, *p value* = 0.5403). The jackknife method showed that the loci standard error of *F*_IS_ was 0.055 and was 27 times bigger than that for *F*_ST_ (0.002). The Micro-Checker analysis suggested the presence of null alleles at four loci (*Aria2*, *Aria3*, *Aria4,* and *Aria14*). According to the criteria described in De Meeûs^30^ (see “Methods” section), null alleles only explained partly (if any) the observed heterozygote deficit.

To check the result stability again, the *F*_IS_ computed for the dataset that included also Group A (complete dataset) was compared with the one obtained without it (Group B) (Supplementary Table S1). The *F*_IS_ was bigger in the complete dataset than in Group B alone (*F*_IS_ = 0.079 and *F*_IS_ = 0.057, respectively), and the difference was significant (*p value* = 0.0486 unilateral Wilcoxon signed rank test). This translated into a Wahlund effect when Group A individuals were included in the data. These individuals were thus kept excluded.

### Regression approach

A Principal Component Analysis (PCA) was undertaken with PCAGen (developed by J. Goudet, freely available at https://www2.unil.ch/popgen/softwares/pcagen.htm). The first two axes were significant using the broken stick criterion but not by permutation testing (*p value* = 0.2187 for axis 1 and *p value* = 0.1045 for axis 2) (see “Methods” section). Axis 1 and axis 2 represented 31% and 23% of the total inertia, respectively. We undertook two generalized linear model (glm) with the coordinates of subsamples at these axes (see “Methods” section). For the first axis, the minimum model, after a stepwise procedure, was: axis1 ~ hillside + latitude + longitude + latitude:hillside. For this axis, none of the included variables played a significant role (*p value* > 0.05 for all tests). For axis 2, the minimum model was: axis2 ~ hillside + altitude + latitude + longitude. Altitude and hillside played a significant role (*p value*s < 0.001) and represented 51% and 15% of the total deviance, respectively (Table 1).

**Table 1:** Minimum model obtained for the regression of PCA axis 2 coordinates of *Phlebotomus ariasi* sampling sites.

Variables	Sum^2^	Pseudo *R*^2^	*p* *value*
Altitude	0.110675	0.5175	0.0006
Hillside	0.023237	0.1525	0.0002
Longitude	0.011227	0.0769	0.1663
Latitude	0.001147	0.0361	0.1430
All		0.7830	

The sum of squares (Sum^2^), the proportion of deviance explained by the model and the proportion of deviance explained for each explanatory variable (Pseudo *R*^2^) are given. The *p value*s were obtained after *F* tests.

### Isolation by altitude distance

As showed above, latitude and longitude had a weak effect on the genetic structure. Geographic parameters were thus removed from the model and isolation by altitudinal distance was tested using the Mantel test for each hillside separately (South–East and North–West, two tests).

When individuals were grouped by altitude levels (see Table 2 and “Methods” section), isolation by altitude distance was significant for the Northern (*p value* = 0.00605) and marginally non-significant for the Southern hillside (*p value* = 0.07345). When combined with the generalized binomial procedure^31^, computed with the MultiTest V1^32^, isolation by altitude appeared highly significant (*p value* = 0.0054).

**Table 2:** Sampling stations (ST) in the study area.

Station	UTM E	UTM N	HO	Alt	HG	Biotope characteristics	N
ST01	31T554956	31T4868394	SE	228	A1	Hamlet	2
ST02	31T554800	31T4868471	SE	244	A1	Hamlet	42
ST03	31T554210	31T4869275	SE	321	A2	Hamlet/Hutch	56
ST04	31T554302	31T4869424	SE	322	A2	Hamlet outskirts	148
ST05	31T554343	31T4869526	SE	341	A2	Kennel	20
ST06	31T554175	31T4869464	SE	354	A2	Hamlet	121
ST07	31T552692	31T4869246	NW	603	A3	Hamlet	9
ST08	31T552715	31T4869102	NW	603	A3	Hamlet	70
ST09	31T552614	31T4868909	NW	573	A3	Countryside	121
ST10	31T552143	31T4869102	NW	539	A3	Countryside	126
ST11	31T551130	31T4868999	NW	417	A4	Countryside	166
ST12	31T550944	31T4869286	NW	397	A4	Countryside	65
ST13	31T550925	31T4869586	NW	362	A4	Countryside	126
ST14	31T550354	31T4869799	NW	343	A5	Countryside	9
ST15	31T549998	31T4870371	NW	282	A5	Hamlet outskirts	116
ST16	31T549453	31T4870290	NW	255	A5	Hamlet	21
ST17	31T549306	31T4870270	NW	245	A5	Hamlet/Sheep barn	35

Station names, Universal Transverse Mercator (UTM) coordinates, hillside orientation (HO) (SE: South–East; NW: North–West), altitude (Alt, in m), hillside group (HG), biotope characteristics, and number of genotyped sand flies (N) are indicated. A1 (Southern hillside, 100–300 m), A2 (Southern hillside, 300–500 m), A3 (Northern hillside, > 500 m), A4 (Northern hillside, 300–500 m), A5 (Northern hillside, 100-300 m).

### Effective population sizes and migration

The average effective population size (*N*_*e*_) was 69 individuals (range: 39 to 98) across subsamples and methods (see “Methods” section). *N*_*e*_ was not correlated with altitude (Spearman's *ρ* = 0.2216, *p value* = 0.7864). There was no effect of hillside (Kruskal–Wallis *p value* = 0.6242). Nei's unbiased estimator of genetic diversities^33^ was *H*_S_ = 0.52 and *H*_T_ = 0.524 for the subsamples and total sample, respectively, with Meirmans and Hedrick's *G*_ST_’’ = 0.017^34^, and then *N*_e_*m* = (1 − *G*_ST_’’)/4 *G*_ST_’’ = 14 (immigrants per generation in each subpopulation assuming an island model of migration). The correlation between Nei's *G*_ST_ and *H*_S_ was strongly negative (rho = − 0.94, *p value* = 0.0083). Therefore, according to Wang’s criterion^35^, it is more accurate using *F*_ST_. *F*_ST_ with the ENA correction for null alleles and 95% confidence intervals (95% CI) after 5,000 bootstraps over loci were computed with FreeNA^36^. This provided *F*_ST_ = 0.014 (95% CI = [0.006, 0.023]), with a corresponding *N*_*e*_*m* = 18 (95% CI [10, 40]).

## Discussion

### Locus selection

In this study, using 11 microsatellite markers, we investigated the genetic structure of *Ph. ariasi* populations in 1,253 individuals collected in 17 stations in the South of France (Supplementary Table S1). Different analyses (Micro-Checker, LD, *F*_IS_ and *F*_ST_ variance) allowed identifying loci with technical problems and/or loci that may not be neutral concerning natural selection. Consequently, we removed five of the eleven loci for various reasons including the presence of incurable stutter artifacts (*Aria10* and *Aria15*), absence of polymorphism (*Aria1*), or non-neutral evolution (*Aria11* and *Aria12*). One locus with SAD could be corrected.

### Presence of outlier individuals

The Bayesian analyses (DAPC and BAPS) for all remaining loci (*Aria2*, *Aria3*, *Aria4*, *Aria5*, *Aria13,* and *Aria14*) confirmed the 22 outliers (Group A). These outliers were morphologically similar to *Ph. ariasi* and displayed 99–100% *cyt*b sequence similarities. A previous study, based on chromatographic analysis of cuticular components, provided evidence that there are two distinct *Ph. ariasi* populations in our study area: one predominantly sylvatic, and the second one domestic^19^. However, in our study, there was no geographical distribution difference between individuals from Group A and Group B.

This finding could reflect the existence of cryptic species, as already suspected or reported for other sand fly species (e.g., *Lutzomyia longipalpis*^37,38^, *Lutzomyia umbratilis*^39^, and *Sergentomyia bailyi*^40^). However, the distinction between Group A and Group B mainly depends on a single genetic marker (*Aria1*), which is fixed for different alleles in each group, while the other markers display a rather weak, though significant, signal. This result is supported by the dendrogram as the same tree was obtained with 6 or 10 loci (excluding *Aria1*). Additional molecular and biological studies will be necessary to test the cryptic species hypothesis (vector competence, interbreeding, hosts and niche preferences, behavior, etc.). As the taxonomic status of Group A individuals could not be elucidated, they were removed from the analyses.

### Genetic structuring in ecotypes

We observed an important and significant heterozygote deficit, instead of the heterozygote excess expected for dioecious populations with random mating^41^. We found no evidence of Wahlund effect with the LD-based method described by Manangwa et al.^42^. Consequently, the heterozygote deficit could (non-exclusively) be explained by (1) null alleles; (2) allelic dropout; or (3) positive assortative mating. In the last case, this would require a mutual attraction of sexual partners based on a sufficient proportion of the genome to allow the hitchhiking of microsatellite markers, which are theoretically non-coding DNA sequences.

The glm approach showed a strong influence of altitude and hillside, and a weak (if any) influence of geographic distances on the genetic data. The proportion of deviance (67%) explained by altitude and hillside suggested the existence of ecotypes in this *Ph. ariasi* population.

The very strong migration rate estimated in our study (more than 25% of the effective subpopulation size) is hard to reconcile with the emergence of genetically distinct ecotypes. Different scenarios can explain ecotype structuring despite the strong migration rate: (1) the death of most immigrants before they can reproduce (unlikely scenario), and (2) significant assortative mating that can also explain the heterozygote deficit observed in our data (see above). The rate of codominant assortative mating based on genes homogeneously distributed in the genome can be approximated with the same equation as for selfing, as described in Hartl et al.^43^ (page 272), with the equation *a* ≈ 2*F*_IS_/(1 + *F*_IS_). With a *F*_IS_ = 0.057, the assortative mating in our population would be ≈ 0.11 (≈ 11% of zygotes produced).

It is worth noting that previous research on the same species in the same study area highlighted differences in wing phenotypes according to altitude and hillside^25^. It has been demonstrated that wing configuration is associated with wing beating frequency and mate recognition^44,45^. These features would lead to a preferential choice of partners that could explain assortative mating and ecotype structuring. More studies are necessary to investigate the link between phenotypic (wing configuration) and genetic (microsatellite) diversity.

### Ecological and epidemiological consequences of this genetic structuration

This structuring has undoubtedly implications on the ecology and evolution of sand flies. This structure might favor the global stability of their populations. Indeed, local environmental changes would have no or low effect on the overall population because the ecotypes would be affected independently. The high levels of migration rates associated with the well-known low capacity of flying of sand flies would help to colonize and recolonize at small-scale environments^46^. It also could explain the global but slow (compared with that of invasive mosquitoes, such as *Aedes albopictus*) geographical expansion of sand flies observed in France^17^.

This particular population structure can also influence *Leishmania* transmission. Previous studies suggested the existence of different imbricated *Leishmania* transmission cycles at very small scales^47,48^. However, due to the low flying capacities of sand flies^46,49^, migrants are expected to disperse mainly over short distances. Therefore, the spread and increase of *L. infantum* cases in France might be mainly due to the movement of infected hosts.

This study demonstrates for the first time that *Ph. ariasi* presents a genetic structure in ecotypes. These data highlight the necessity to consider sand fly populations at small and specific scales to determine their ecology and its impact on *Leishmania* transmission. This structure may explain the long-term stability of sand fly populations.

Not many papers compare available clustering algorithms to date and it is thus hard to really understand when and why such algorithms will converge or diverge in the best partition they offer. For some attempt comparisons see: Latch et al.^50^, Kaeuffer et al.^51^, Frantz et al.^52^, Blair et al.^53^, Bohling et al.^54^, Manangwa et al.^42^.

This type of study needs to be extended to other sand fly species. Indeed, the large diversities of sand fly populations worldwide may correlate with different ecological vector capacities and sensitivity to control measures.

## Methods

### Study area

**Figure 5:**
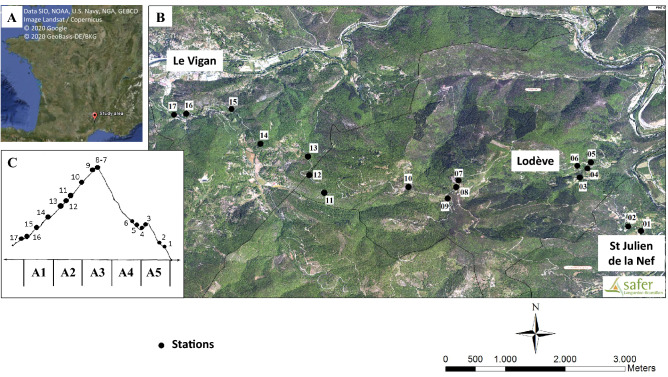
Localization (**A**), map (**B**) and profile (**C**) of the study area. Red dots and numbers indicate the sampling stations. A1 (Northern hillside, 100–300 m), A2 (Northern hillside, 300–500 m), A3 (Northern hillside, > 500 m), A4 (Southern hillside, 300–500 m), and A5 (Southern hillside, 300–100 m). The map came from three sources ([**A**] Google Earth, [**B**] SAFER and [**C**] adapted from Rioux et al.^3^ "Creative Commons license") that were combined under Adobe Illusrator.

The field study was performed in the South of France, on the “massif de l’Oiselette” hill situated between the “Hérault” (Ganges, Hérault) and “Arre” (Le Vigan, Gard) valleys. Sand flies were sampled along a 14 km transect from the “Saint Julien de la Nef” to “Le Vigan” villages, including “Roquedur-le-haut” (at 601 m above sea level) (Fig. 5; Table 2). This region has a Mediterranean sub-humid climate^55^, and is characterized by the presence of plant species typical of scrubland habitats^5^.

The study area was divided in two hillsides with a South–East and North–West orientation, like in previous works performed in this area^3,24,25^. Stations were selected based on the paper of Rioux et al.^3^, first to allow the comparison with the data obtained 30 years ago in terms of species distribution, density and abundance^24^, second because these stations were distributed along the 14 km transect and represented the altitude and hillside diversity necessary for our population genetics study. Station 7 and Station 8 were not present in Rioux et al.^3^. These two stations are transitional between the two hillsides, and were thus added in the present study to obtain information on the consequences of transitional ecosystems. The stations were grouped according to altitude and hillside (see Fig. 5; Table 2). This area is characterized by the presence of various domestic animals, such as chicken, sheep, ducks, geese, horses, rabbits, cats, dogs, and also many different wild animals (wild boars, foxes, rodents, lizards, birds, etc.). These animals represent potential sand fly hosts. Moreover, cases of canine leishmaniasis were observed during the collection period (J.P. personal observation).

### Sand fly collection and identification

Sand flies were collected monthly using 3,589 sticky traps (20 × 20 cm white paper covered with castor oil, as described by Alten et al.^56^ and Ayhan et al.^57^) between May and September 2011. Seventeen localities (sampling stations) were sampled (Fig. 5; Table 2) with a mean of 189 sticky traps per station, in various biotopes, inside and around human dwellings and animal sheds, close to the vegetation, and in wall crevices. Each trap was collected after 2 days.

In total, 1,253 sand flies were captured and transferred individually into 1.5 mL Eppendorf tubes with 96% ethanol and labeled. Prior to mounting, the sand fly head, genitalia and wings were removed. Heads and genitalia were cleared in Marc-André solution (chloral hydrate/acetic acid) and mounted in chloral gum^46^. Each individual specimen was identified on the basis of the morphology of the pharynges and/or male genitalia or female spermathecae, using the keys of Abonnenc^46^, Lewis^6^ and Killick-Kendrick et al.^58^.

### DNA extraction

Sand fly DNA was extracted using the Chelex method^59^ described in Prudhomme et al.^27^. Extraction was performed at the UMR “Unité des Virus Emergents” (UVE, IHU Méditerranée Infection, Marseille, France). Each entire sand fly was ground using a Mixer Mill MM300 (QIAGEN, Venlo, Netherlands) with one 3-mm tungsten bead in 200 μL Eagle’s Minimal Essential Medium at a frequency of 30 cycles s^−1^ for 3 min. A volume of 140 μL of each sample was then used for DNA purification by adding the Chelex resin suspension^59^ or by using the Eppendorf epMotion 5075 working station and the Macherey–Nagel NucleoSpin 96 Virus kit.

### DNA amplification and genotyping

Based on the microsatellite position in the sequence and the repeated pattern structure**,** 11 of the most polymorphic loci were selected among the previously described 16 microsatellite markers for *Ph. ariasi*^27^. Each 25 μL reaction mix included 1 pmol of forward (labeled with the fluorochrome FAM, ATT0565 or HEX) and reverse primers, 5 ng of sand fly DNA sample, 6 pmol of dNTP mix, 2.5 μL of 10X buffer, and 0.25 μL of Taq polymerase (ROCHE DIAGNOSTICS, 5 UI/μL). DNA was amplified in a thermal cycler using the following conditions: initial denaturation step at 95 °C for 10 min, followed by 40 cycles at 95 °C for 30 s, the specific annealing temperature of each locus^27^ for 30 s, 72 °C for 1 min, and a final extension step at 72 °C for 10 min. For genotyping, 1 μL of PCR product was added into a standard loading mix (0.5 μL of the internal GeneScan 500LIZ dye size standard and 12.5 μL of Hi-Di formamide) (both from APPLIED BIOSYSTEMS) and sequenced on an ABI Prism 3130 Genetic Analyzer (APPLIED BIOSYSTEMS) automated sequencer. Profiles were read and analyzed using GENEMAPPER 4.0 [APPLIED BIOSYSTEMS, Foster City (CA)].

DNA from a subset of *Ph. ariasi* samples was used to check the species identification (see “Results” section) by amplifying a *cyt*b gene fragment using the primers N1N-PDR [5′-CA(T/C) ATT CAA CC(A/T) GAA TGA TA-3′] and C3B-PDR [5′-GGT A(C/T)(A/T) TTG CCT CGA (T/A)TT CG(T/A) TAT GA-3′], according to a previously published protocol^60,61^ and the following conditions: initial denaturation at 94 °C of 3 min; 5 cycles of denaturation (94 °C for 30 s), annealing (40 °C for 60 s), and extension (68 °C for 60 s), followed by 40 cycles of denaturation (94 °C for 60 s), annealing (44 °C for 60 s) and extension (68 °C for 60 s), and a final extension (68 °C for 10 min). Direct sequencing in both directions was performed by Eurofins Genomics.

### Data analysis

Microsatellite raw data were formatted for CREATE^62^ that allowed their transformation into the formats needed for the different analyses. Samples with more than 50% of missing data were removed from the analyses.

#### Bayesian clustering

Several Bayesian clustering analyses were carried out. To validate the sand fly species identification, the first analysis was based on the 11 selected loci and included all sand fly samples. Then, to study the organization of *Ph. ariasi* populations, data were analyzed at different scales: hillside, altitude, and station. This analysis was performed with the loci selected after Linkage Disequilibrium (LD) and *F-*statistics analyses (see “Results” section).

As the clustering method accuracy can vary depending on the statistical properties of specific software programs and datasets^29,42,63^, three different Bayesian clustering methods were used. First, a discriminant analysis of principal components (DAPC)^64^ was performed using the *adegenet*^65^ package for R^66^. This was followed by a Bayesian Analysis of Population Structure (BAPS; admixture model^67,68^, maximum number of clusters: 35, repetition: 50; that is freely available at https://www.helsinki.fi/bsg/software/BAPS/), and a STRUCTURE (version 2.3.4) analysis^69^ (burning period: 10,000, number of clusters from 1 to 35, with the admixture model). STRUCTURE HARVESTER vA.2^70^ was used to visualize the STRUCTURE analysis results, examine the ad hoc ΔK statistic, and determine the optimal number of clusters.

Finally, a dendrogram (NJTree) was built with MEGA 7^71^ from a Cavalli-Sforza and Edward's chord distance (*D*_CSE_)^72^ matrix as recommended^73^, between subsamples defined as combinations of the group obtained by Bayesian clustering and sampling stations. Because null alleles were suspected to occur, *D*_CSE_ was computed with the INA correction with FreeNA^36^, after recoding missing data into null homozygotes following authors' recommendation.

#### Linkage disequilibrium and F-statistics

LD between each locus pair was tested with the *G*-based permutation test with 10,000 randomizations. This test was performed with F_STAT_ 2.9.4, an updated version of F_STAT_ 2.9.3^74^ available at https://www.t-de-meeus.fr/ProgMeeusGB.html. This procedure is the most powerful for testing LD across different subsamples^32^. There are as many *p value*s as locus pairs. Then, the False Discovery Rate (FDR) correction for multiple non-independent tests described by Benjamini et al.^75^ was applied with R 3.5.1^66^.

Wright's *F* statistics^76^ were estimated with the Weir and Cockerham's unbiased estimators^77^. Significant departure from 0, for *F* statistics, was tested by randomizing alleles between individuals within subsamples (deviation from the local random mating test) or individuals between subsamples within the total sample (population subdivision test). The *p value* corresponded to the number of times a statistic measured in randomized samples was as big as (or bigger than) the observed one (unilateral tests). For local panmixia, the statistic used was *f* (Weir and Cockerham's *F*_IS_ estimator). To test for subdivision, the *G-*based test^78^ was used. According to De Meeûs et al.^32^, the *G-*based test is the most powerful procedure when combining tests across loci.

The 95% confidence intervals (CI) of *F*-statistics were computed using the jackknife over populations method for each locus or 5,000 bootstraps over loci for the averages, as described in De Meeûs et al.^79^. Parameter estimates, testing, jackknife and bootstrap computations were done with F_STAT_ 2.9.4.

The determination procedure described by De Meeûs^30^ and Manangwa et al.^42^ was used to discriminate demographic from technical causes of significant homozygote excess and LD. In the case of null alleles, *F*_IS_ and *F*_ST_ artificially increase and a positive correlation is expected between the statistics, *F*_IS_ standard error is at least twice that of *F*_ST_, and a positive correlation is also expected between *F*_IS_ and the number of missing data (putative null homozygotes). Correlations were tested with the unilateral Spearman’s rank correlation test in R 3.5.1^66^. The frequency of null alleles was assessed with Micro-Checker v2.2.3^80^ using Brookfield's second method^81^.

The presence of stutter artifacts at each locus in each subsample was evaluated with Micro-Checker v2.2.3^80^. A unilateral exact binomial test was used with R 3.5.1^66^ to determine whether the observed proportion of significant stutter artifacts was greater than the expected 5% under the null hypothesis.

Short Allele Dominance (SAD) was assessed with the method described by Manangwa et al.^42^. The correlation between allele size and *F*_IT_ was tested with the unilateral Spearman’s rank correlation test in R 3.5.1^66^. In the case of SAD, a negative correlation is expected between allele size and *F*_IT_^42^.

In the case of significant stutter artifacts or SAD, the incriminated loci were corrected using the method described by De Meeûs et al.^29^. Stuttering was addressed by pooling alleles close in size. To avoid a spurious increase of heterozygosity, each pooled group contained at least one frequent allele (e.g., with *p value* ≥ 0.05). SAD was addressed by going back to the chromatograms of homozygous individuals and trying to find a larger size micro-peak that could have been missed in the first reading.

In some instances, *F*_IS_ values were compared between subsample groups using the Wilcoxon signed rank test for paired data (the locus was the pairing unit).

#### Role of environmental factors on sand fly structuring

A principal component analysis (PCA) was done with PCAGen 1.2.1 (developed by J. Goudet, freely available at https://www2.unil.ch/popgen/softwares/pcagen.htm). The significance of the first axes was tested using the broken stick criterion^82^ and 10,000 permutations of individuals across subsamples. The metric of each axis divided by the total genetic diversity corresponded to Weir & Cockerham's Theta (*F*_ST_ estimator) (Goudet’s personal communication). The coordinates of subsamples for each significant axis were used as the response variable of generalized linear models (glm). General models were as follows: axis *i* ~ latitude + longitude + altitude + hillside + latitude: hillside + longitude:hillside + altitude:hillside (where *i* corresponded to the significant axes; latitude and longitude are the latitudinal and longitudinal GPS coordinates in decimal degrees; altitude is the altitude in meters; hillside: south–east or north–west; and "X:Y" represents the interaction between the explanatory variables X and Y). All glm's were done using R version 3.5.1^66^, with the package *rcmdr* (R-commander)^83,84^. Model selection was performed using a forward stepwise model selection procedure and the Akaike Information Criterion^85^.

The influence of different factors (including interactions) was tested by examining the differences between models (complete, additive, and with one variable) with analyses of variances. As the entry order of explanatory variables matters in R analyses, the mean partial *R*^2^ of each variable was calculated across all possible models.

#### Isolation by distance

Geographic distances were computed with Genepop version 4.7.0^86^ using the Euclidian distance computed with the Universal Transverse Mercator (UTM) coordinates (Table 2). Genetic distances were estimated with the Cavalli-Sforza and Edwards chord distance^72^ *D*_CSE_, the most powerful method to detect isolation by distance with microsatellite markers in most situations^87^. Due to the presence of missing data, data were converted into the FreeNA format to compute *D*_CSE_ between each station pair with the ENA correction^36^ that provides a very good correction for the isolation by distance regression slope in the case of null alleles^87^. As these station pair ended into a non‐squared matrix that could not be handled by Genepop, the relationships between genetic and geographic distances were tested with a Mantel test (10^4^ permutations)^88^ in F_STAT_ 2.9.4. As the Mantel test in F_STAT_ is bilateral by default, the *p value* was halved in the case of positive slope, or computed as: “1-(1-bilateral *p values*)/2” in the case of negative slope, to obtain unilateral *p value*s for a positive slope.

The relationships between genetic and altitudinal distances were also tested in the conditions described above. To avoid any interaction, the analyses for the South and North hillsides were done separately. The two *p value*s obtained were then combined with the generalized binomial test^31^, with MultiTest^32^, taking into account all tests as recommended when the number of combined tests *k* < 4^89^.

#### Effective population sizes and migration

Four different methods were used to estimate the effective population sizes (*N*_*e*_). The results obtained with these methods were averaged and weighted by the number of times a usable value (after removal of “infinity” results) was obtained. To obtain a range of possible *N*_*e*_ values, the same approach was performed for the minimum and maximum values obtained with each method.

First, NeEstimator v2^90^ was used with the LD method^91,92^ that applies a correction for missing data^93^ and the molecular co-ancestry method^94^. The LD method uses several threshold allele frequencies (0.05, 0.02, 0.01, and all alleles) to compute *N*_*e*_. The average across the different values obtained with these frequencies was computed.

The intra- and inter-loci correlation method was also used to compute *N*_*e*_^95^ using Estim v2.2^96^ (available at: https://www.t-demeeus.fr/ProgMeeusGB.html).

Finally, the heterozygote-excess method (expected in dioecious populations) described by Balloux^41^ was used for each locus that displayed heterozygote excess, as follows: *N*_*e*_ = [− 1/(2 *F*_IS_)] −*F*_IS_/(1 + *F*_IS_).

To determine the number of immigrants, the standardized differentiation index described by Meirmans and Hedrick was computed to correct for polymorphism excess: *G*_ST_" = n × (*H*_T_ − *H*_S_)/[(n × *H*_T_ − *H*_S_) × (1 − *H*_S_)]^34^, where *H*_T_ and *H*_S_ are Nei's unbiased estimates of genetic diversity in the total sample or within subsamples, respectively^33^, and *n* is the number of subsamples. Genetic diversities were estimated with F_STAT_ 2.9.4. Assuming an island model, this value was then used to obtain an approximation for the number of immigrants within subpopulations as *N*_*e*_*m* = (1 − *G*_ST_")/(4 × *G*_ST_"). However, according to Wang’s criterion^35^ if Nei’s *G*_ST_^33^ is negatively correlated with *H*_S_, it is wiser to use *F*_ST_ for this computation.

## Supplementary information

Supplementary information

Supplementary Table

## Data Availability

All resources used in this article are provided in the Supporting Information and all the analyses are detailed allowing the assessment or verification of the manuscript’s findings.

## References

[1] Dolmatova AV, Demina NA (1966). Les phlébotomes (Phlebotominae) et les maladies qu'ils transmettent. ORSTOM.

[2] Bichaud L, Souris M, Mary C, Ninove L, Thirion L, Piarroux RP, Piarroux R, De Lamballerie X, Charrel R (2011). Epidemiologic relationship between toscana virus infection and *Leishmania infantum* due to common exposure to *Phlebotomus perniciosus* sandfly vector. PLoS Negl. Trop. Dis..

[3] Rioux J-A, Killick-Kendrick R, Perieres J, Turner D, Lanotte G (1980). Ecologie des Leishmanioses dans le sud de la France. 13. Les sites de "flanc de coteau", biotopes de transmission privilégiés de la Leishmaniose viscérale en Cévennes. Ann . Parasitol. Hum. Comp..

[4] Rioux J-A, Carron S, Dereure J, Perieres J, Zeraia L, Franquet E, Babinot M, Gallego M, Prudhomme J (2013). Ecology of leishmaniasis in the South of France. 22. Reliability and representativeness of 12 *Phlebotomus ariasi*, *P. perniciosus* and *Sergentomyia minuta* (Diptera: Psychodidae) sampling stations in Vallespir (eastern French Pyrenees region). Parasite.

[5] Rioux J-A, Golvan Y, Croset H, Tour S, Hovin R, Abonnenc E, Petitdidier M, Vollhardt Y, Dedet JP, Albaret JL (1969). Epidémiologie des leishmanioses dans le Sud de la France. Monogr. l'Inst. Natl. Santé Rech. Méd..

[6] Lewis DJ (1982). A taxonomic review of the genus *Phlebotomus* (Diptera: Psychodidae). Bull. Br. Museum.

[7] Rossi E, Rinaldi L, Musella V, Veneziano V, Carbone S, Gradoni L, Cringoli G, Maroli M (2007). Mapping the main *Leishmania* phlebotomine vector in the endemic focus of the Mt. Vesuvius in southern Italy. Geospat. Health.

[8] Ballart C, Barón S, Alcover MM, Portus M, Gallego M (2012). Distribution of phlebotomine sand flies (Diptera: Psychodidae) in Andorra: First finding of *P. perniciosus* and wide distribution of *P. ariasi*. Acta Trop..

[9] Ballart C (2014). Importance of individual analysis of environmental and climatic factors affecting the density of *Leishmania *vectors living in the same geographical area: The example of *Phlebotomus ariasi* and *P. perniciosus* in northeast Spain. Geospat. Health.

[10] Boussaa S, Neffa M, Pesson B, Boumezzough A (2010). Phlebotomine sandflies (Diptera: Psychodidae) of southern Morocco: Results of entomological surveys along the Marrakech-Ouarzazat and Marrakech-Azilal roads. Ann. Trop. Med. Parasitol..

[11] Franco F, Morillas-Marquez F, Barón S, Morales-Yuste M, Gálvez R, Díaz-Sáez V, Pesson B, Alves-Pires C, Depaquit J, Molina R (2010). Genetic structure of *Phlebotomus* (*Larroussius*) *ariasi* populations, the vector of *Leishmania infantum* in the western Mediterranean: Epidemiological implications. Int. J. Parasitol..

[12] Ready P (2010). Leishmaniasis emergence in Europe. Euro Surveill..

[13] Branco S, Alves-Pires C, Maia C, Cortes S, Cristovão J, Gonçalves L, Campino L, Afonso M (2013). Entomological and ecological studies in a new potential zoonotic leishmaniasis focus in Torres Novas municipality, Central Region, Portugal. Acta Trop..

[14] Barón SD, Morillas-Márquez F, Morales-Yuste M, Díaz-Sáez V, Irigaray C, Martín-Sánchez J (2011). Risk maps for the presence and absence of *Phlebotomus perniciosus* in an endemic area of leishmaniasis in southern Spain: Implications for the control of the disease. Parasitology.

[15] Boudabous R, Amor S, Khayech F, Marzouk M, Bdira S, Mezhoud H, Azaiez R, Sfar M, Babba X (2009). The phlebotomine fauna (Diptera: Psychodidae) of the eastern coast of Tunisia. J. Med. Entomol..

[16] European Centre for Disease Prevention and Control E. Phlebotomine sand flies maps [internet] 2019 [10/01/19]. https://www.ecdc.europa.eu/en/disease-vectors/surveillance-and-disease-data/phlebotomine-maps.

[17] Dedet J-P (2020). Les leishmanioses en France métropolitaine. BEH Hors-Sér..

[18] Depaquit J, Grandadam M, Fouque F, Andry P-E, Peyrefitte C (2010). Arthropod-borne viruses transmitted by Phlebotomine sandflies in Europe: A review. Euro Surveill..

[19] Kamhawi S, Molyneux D, Killick-Kendrick R, Milligan P, Phillips A, Wilkes T, Killick-Kendrick M (1987). Two populations of *Phlebotomus ariasi* in the Cévennes focus of leishmaniasis in the south of France revealed by analysis of cuticular hydrocarbons. Med. Vet. Entomol..

[20] Pesson B, Wallon M, Floer M, Kristensen A (1991). Étude isoenzymatique de populations méditerranéennes de phlébotomes du sous-genre *Larroussius*. Parassitologia.

[21] Ballart C, Pesson B, Gallego M (2018). Isoenzymatic characterization of *Phlebotomus ariasi* and *P. perniciosus* of canine leishmaniasis foci from Eastern Pyrenean regions and comparison with other populations from Europe. Parasite..

[22] Martin-Sanchez J, Gramiccia M, Pesson B, Morillas-Marquez F (2000). Genetic polymorphism in sympatric species of the genus *Phlebotomus*, with special reference to *Phlebotomus perniciosus* and *Phlebotomus longicuspis* (Diptera, Phlebotomidae). Parasite.

[23] Mahamdallie SS, Pesson B, Ready PD (2010). Multiple genetic divergences and population expansions of a Mediterranean sandfly, *Phlebotomus ariasi*, in Europe during the Pleistocene glacial cycles. Heredity.

[24] Prudhomme J, Rahola N, Toty C, Cassan C, Roiz D, Vergnes B, Thierry M, Rioux J-A, Alten B, Sereno D (2015). Ecology and spatiotemporal dynamics of sandflies in the Mediterranean Languedoc region (Roquedur area, Gard, France). Parasit. Vectors.

[25] Prudhomme J, Cassan C, Hide M, Toty C, Rahola N, Vergnes B, Dujardin J-P, Alten B, Sereno D, Banuls A-L (2016). Ecology and morphological variations in wings of *Phlebotomus ariasi* (Diptera: Psychodidae) in the region of Roquedur (Gard, France): A geometric morphometrics approach. Parasit. Vectors.

[26] Lachaud L, Dedet J, Marty P, Faraut F, Buffet P, Gangneux J, Ravel C, Bastien P (2013). Surveillance of leishmaniases in France, 1999 to 2012. Euro Surveill..

[27] Prudhomme J, Toty C, Kasap O, Rahola N, Vergnes B, Maia C, Campino L, Antoniou M, Jimenez M, Molina R (2015). New microsatellite markers for multi-scale genetic studies on *Phlebotomus ariasi* Tonnoir, vector of *Leishmania infantum* in the Mediterranean area. Acta Trop..

[28] Wattier R, Engel CR, Saumitou-Laprade P, Valero M (1998). Short allele dominance as a source of heterozygote deficiency at microsatellite loci: Experimental evidence at the dinucleotide locus Gv1CT in *Gracilaria gracilis* (Rhodophyta). Mol. Ecol..

[29] De Meeûs T, Chan CT, Ludwig JM, Tsao JI, Patel J, Bhagatwala J, Beati L. Deceptive combined effects of short allele dominance and stuttering: An example with *Ixodes scapularis*, the main vector of Lyme disease in the U.S.A. peerreviewed and recommended by PCI Evolutionary Biology. 2019.

[30] De Meeûs T (2018). Revisiting, *F*_IS_, *F*_ST_, Wahlund Effects, and Null Alleles. J. Hered..

[31] Teriokhin AT, De Meeûs T, Guegan JF (2007). On the power of some binomial modifications of the Bonferroni multiple test. J. Gener. Biol..

[32] De Meeûs T, Guégan J-F, Teriokhin AT (2009). MultiTest V.1.2., a program to binomially combine independent tests and performance comparison with other related methods on proportional data. BMC Bioinform..

[33] Nei M, Chesser RK (1983). Estimation of fixation indices and gene diversities. Ann. Hum. Genet..

[34] Meirmans PG, Hedrick PW (2011). Assessing population structure: *F*_ST_ and related measures. Mol. Ecol. Resour..

[35] Wang J (2015). Does *G*_ST_ underestimate genetic differentiation from marker data?. Mol. Ecol..

[36] Chapuis MP, Estoup A (2007). Microsatellite null alleles and estimation of population differentiation. Mol. Biol. Evol..

[37] Maingon R, Ward R, Hamilton J, Noyes H, Souza N, Kemp S, Watts P (2003). Genetic identification of two sibling species of *Lutzomyia longipalpis* (Diptera: Psychodidae) that produce distinct male sex pheromones in Sobral, Ceará State, Brazil. Mol. Ecol..

[38] Bauzer LG, Souza NA, Maingon RD, Peixoto AA (2007). *Lutzomyia longipalpis* in Brazil: A complex or a single species? A mini-review. Mem. Inst. Oswaldo Cruz..

[39] Scarpassa VM, Alencar RB (2012). *Lutzomyia umbratilis*, the main vector of *Leishmania guyanensis*, represents a novel species complex?. PLoS One.

[40] Tharmatha T, Gajapathy K, Ramasamy R, Surendran SN (2016). Morphological and molecular identification of cryptic species in the *Sergentomyia bailyi* (Sinton, 1931) complex in Sri Lanka. Bull. Entomol. Res..

[41] Balloux F (2004). Heterozygote excess in small populations and the heterozygote-excess effective population size. Evolution.

[42] Manangwa O, De Meeus T, Grebaut P, Segard A, Byamungu M, Ravel S (2019). Detecting Wahlund effects together with amplification problems: Cryptic species, null alleles and short allele dominance in *Glossina pallidipes* populations from Tanzania. Mol. Ecol. Resour..

[43] Hartl DL, Clarck AG (1989). Principles of Population Genetics.

[44] Araki AS, Ferreira G, Mazzoni CJ, Souza NA, Machado RC, Bruno RV, Peixoto AA (2013). Multilocus analysis of divergence and introgression in sympatric and allopatric sibling species of the *Lutzomyia longipalpis* complex in Brazil. PLoS Negl Trop Dis..

[45] Kyriacou C (2014). Sex and rhythms in sandflies and mosquitoes: an appreciation of the work of Alexandre Afranio Peixoto (1963–2013). Infect. Genet. Evol..

[46] Abonnenc E. Les phlébotomes de la région éthiopienne (Diptera, Psychodidae): Cahiers de l'ORSTOM, série Entomologie médicale et Parasitologie; 1972 01/01. 239.

[47] Rougeron V, Bañuls AL, Carme B, Simon S, Couppie P, Nacher M, Hide M, De Meeûs T (2011). Reproductive strategies and population structure in *Leishmania*: Substantial amount of sex in *Leishmania Viannia guyanensis*. Mol. Ecol..

[48] Rougeron V, De Meeûs T, Hide M, Le Falher G, Bucheton B, Dereure J, El-Safi SH, Dessein A, Bañuls A-L (2011). Multifaceted population structure and reproductive strategy in *Leishmania donovani* complex in one Sudanese village. PLoS Negl. Trop. Dis..

[49] Rioux J-A (1979). Ecologie des Leishmanioses dans le sud de la France. 12. Dispersion horizontale de *Phlebotomus ariasi* Tonnoir, 1921. Experiences préliminaires. Ann. Parasitol. Hum. Comp..

[50] Latch EK, Dharmarajan G, Glaubitz JC, Rhodes OE (2006). Relative performance of Bayesian clustering software for inferring population substructure and individual assignment at low levels of population differentiation. Conserv. Genet..

[51] Kaeuffer R, Réale D, Coltman D, Pontier D (2007). Detecting population structure using STRUCTURE software: Effect of background linkage disequilibrium. Heredity.

[52] Frantz AC, Cellina S, Krier A, Schley L, Burke T (2009). Using spatial Bayesian methods to determine the genetic structure of a continuously distributed population: Clusters or isolation by distance?. J. Appl. Ecol..

[53] Blair C, Weigel DE, Balazik M, Keeley AT, Walker FM, Landguth E, Cushman S, Murphy M, Waits L, Balkenhol N (2012). A simulation-based evaluation of methods for inferring linear barriers to gene flow. Mol. Ecol. Resour..

[54] Bohling JH, Dellinger J, McVey JM, Cobb DT, Moorman CE, Waits LP (2016). Describing a developing hybrid zone between red wolves and coyotes in eastern North Carolina, USA. Evol. Appl..

[55] Le DP (1977). bioclimat Mediterraneen: Analyse des formes climatiques par le systeme d'Emberger. Vegetation.

[56] Alten B, Ozbel Y, Ergunay K, Kasap O, Cull B, Antoniou M, Velo E, Prudhomme J, Molina R, Bañuls A (2015). Sampling strategies for phlebotomine sand flies (Diptera: Psychodidae) in Europe. Bull. Entomol. Res..

[57] Ayhan N, Baklouti A, Prudhomme J, Walder G, Amaro F, Alten B, Moutailler S, Ergunay K, Charrel RN, Huemer H (2017). Practical guidelines for studies on sandfly-borne phleboviruses: Part I: Important points to consider ante field work. Vector Borne Zoonot. Dis..

[58] Killick-Kendrick R, Tang Y, Killick-Kendrick M, Sang D, Sirdar M, Ke L, Ashford R, Schorscher J, Johnson R (1991). The identification of female sandflies of the subgenus *Larroussius* by the morphology of the spermathecal ducts. Parassitologia.

[59] Wang Q, Wang X (2012). Comparison of methods for DNA extraction from a single chironomid for PCR analysis. Pak. J. Zool..

[60] Esseghir S, Ready PD, Killick-Kendrick R, Ben-Ismail R (1997). Mitochondrial haplotypes and phylogeography of *Phlebotomus* vectors of *Leishmania major*. Insect. Mol. Biol..

[61] Depaquit J, Leger N, Randrianambinintsoa FJ (2015). Paraphyly of the subgenus *Anaphlebotomus* and creation of *Madaphlebotomus* subg. Nov. (Phlebotominae: *Phlebotomus*). Med. Vet. Entomol..

[62] Coombs JA, Letcher BH, Nislow KH (2008). Create: A software to create input files from diploid genotypic data for 52 genetic software programs. Mol. Ecol. Resour..

[63] Bohling JH, Adams JR, Waits LP (2013). Evaluating the ability of Bayesian clustering methods to detect hybridization and introgression using an empirical red wolf data set. Mol. Ecol..

[64] Jombart T, Devillard S, Balloux F (2010). Discriminant analysis of principal components: A new method for the analysis of genetically structured populations. BMC Genet..

[65] Jombart T (2008). adegenet: A R package for the multivariate analysis of genetic markers. Bioinformatics.

[66] R Development Core Team RT. R: A language and environment for statistical computing. R Foundation for Statistical Computing, Vienna, Austria, 2012. https://www.R-project.org/. 2018.

[67] Corander J, Marttinen P (2006). Bayesian identification of admixture events using multilocus molecular markers. Mol. Ecol..

[68] Corander J, Marttinen P, Siren J, Tang J (2008). Enhanced Bayesian modelling in BAPS software for learning genetic structures of populations. BMC Bioinform..

[69] Pritchard JK, Stephens M, Donnelly P (2000). Inference of population structure using multilocus genotype data. Genetics.

[70] Earl DA, vonHoldt BM (2012). STRUCTURE HARVESTER: A website and program for visualizing STRUCTURE output and implementing the Evanno method. Conserv. Genet. Resourc..

[71] Kumar S, Stecher G, Tamura K (2016). MEGA7: Molecular evolutionary genetics analysis version 7.0 for bigger datasets. Mol. Biol. Evol..

[72] Cavalli-Sforza LL, Edwards AWF (1967). Phylogenetic analysis. Models and estimation procedures. Am. J. Hum. Genet..

[73] Takezaki N, Nei M (1996). Genetic distances and reconstruction of phylogenetic trees from microsatellite DNA. Genetics.

[74] Goudet J (1995). FSTAT (Version 1.2): A computer program to calculate F-statistics. J. Hered..

[75] Benjamini Y, Yekutieli D (2001). The control of the false discovery rate in multiple testing under dependency. Ann. Stat..

[76] Wright S (1965). The interpretation of population structure by F-statistics with special regard to systems of mating. Evolution.

[77] Weir BS, Cockerham CC (1984). Estimating F-statistics for the analysis of population structure. Evolution.

[78] Goudet J, Raymond M, De Meeûs T, Rousset F (1996). Testing differentiation in diploid populations. Genetics.

[79] De Meeûs T, McCoy KD, Prugnolle F, Chevillon C, Durand P, Hurtrez-Boussès S, Renaud F (2007). Population genetics and molecular epidemiology or how to "débusquer la bête". Infect. Genet. Evol..

[80] Van Oosterhout C, Hutchinson WF, Wills DP, Shipley P (2004). MICRO-CHECKER: Software for identifying and correcting genotyping errors in microsatellite data. Mol. Ecol. Notes..

[81] Brookfield JF (1996). A simple new method for estimating null allele frequency from heterozygote deficiency. Mol. Ecol..

[82] Frontier S (1976). Étude de la décroissance des valeurs propres dans une analyse en composantes principales: Comparaison avec le modèle du bâton brisé. J. Exp. Mar. Biol. Ecol..

[83] Fox J, The R (2005). Commander: A basic-statistics graphical user interface to R. J. Stat. Softw..

[84] Fox J (2007). Extending the R Commander by “Plug-In” Packages. R News.

[85] Akaïke H (1974). A new look at the statistical model identification. IEEE Trans. Autom. Control.

[86] Rousset F (2008). GENEPOP'007: A complete re-implementation of the GENEPOP software for Windows and Linux. Mol. Ecol. Resour..

[87] Séré M, Thevenon S, Belem AMG, De Meeus T (2017). Comparison of different genetic distances to test isolation by distance between populations. Heredity.

[88] Mantel N (1967). The detection of disease clustering and a generalized regression approach. Cancer Res..

[89] De Meeus T (2014). Statistical decision from k test series with particular focus on population genetics tools: A DIY notice. Infect. Genet. Evol..

[90] Do C (2014). NeEstimator v2: Re-implementation of software for the estimation of contemporary effective population size (*N*_*e*_) from genetic data. Mol. Ecol. Resour..

[91] Waples RS (2006). A bias correction for estimates of effective population size based on linkage disequilibrium at unlinked gene loci*. Conserv. Genet..

[92] Waples RS, Do C (2008). ldne: A program for estimating effective population size from data on linkage disequilibrium. Mol. Ecol. Resour..

[93] Peel D, Waples RS, Macbeth GM, Do C, Ovenden JR (2013). Accounting for missing data in the estimation of contemporary genetic effective population size (*N*_*e*_). Mol. Ecol. Resour..

[94] Nomura T (2008). Estimation of effective number of breeders from molecular coancestry of single cohort sample. Evol. Appl..

[95] Vitalis R, Couvet D (2001). Estimation of effective population size and migration rate from one- and two-locus identity measures. Genetics.

[96] Vitalis R, Couvet D (2005). Estim 1.0: A computer program to infer population parameters from one- and two-locus gene identity probabilities. Mol. Ecol. Notes.

