# Supplementary material for: Altitude and hillside orientation shapes the population structure of the Leishmania infantum vector Phlebotomus ariasi
Source: Sci Rep. 2020 Sep 2;10:14443. doi: 10.1038/s41598-020-71319-w (PMC7468129; doi:10.1038/s41598-020-71319-w)
Supplement: Supplementary file 1 — Supplementary information [file 41598_2020_71319_MOESM1_ESM.pdf]

**Supplementary File 1.** Mitochondrial cytochrome b gene sequences of eight studied *Phlebotomus ariasi* individuals (#12, 17, 18, 19, 23, 856, 872, and 874).

Altitude and hillside orientation shapes the population structure of the *Leishmania infantum* vector *Phlebotomus ariasi*

Jorian Prudhomme, Thierry De Meeûs, Céline Toty, Cécile Cassan, Nil Rahola, Baptiste Vergnes, Remi Charrel, Bulent Alten4, Denis Sereno, Anne-Laure Bañuls.

**> Sample 12**

Primer N1N-PDR

CNNTAAATTAGGAGGTGTAATTGCACTTGTTATATCAATTGCAATCCTATTTATTCTTCCTATTTTAC
ATGTAAATAAATCTCAAGGATTACAATTTTATCCTCTTAATCAAATCTTATTTTGATATATAGTTATT
ATTATTATTTTATTAACATGAATTGGAGCACGACCAGTTGAATCCCCTTTTATTTTAACAGGACAAA
TTCTTACAGTACTCTACTTCTCATACTATATTTTAAACCCTATAATTTCTAAATTTTGAGATAAATTT
CTAAATTAACCTATTTAGTTAATGAGCTTGATTTAAGCAATTGTTTTGAAAACATTTGATAGAAACT
AAAATTTTCTATTAACTTTACTAATTTTAATTATTATAATAAAATAATTTTTAATCCAATAAAAAAA
ATAATATAACATAATGAAGCAGGTAAATAACTTTTTCAAACTAAATATATTAATTTATCATANCGA
ANTCNN

Primer C3B-PDR

NNTANTTACCTGCTTCATTATGTTATATTATTTTTTTTATTGGATTAAAAATTATTTTATTATAATAA
TTAAAATTAGTAAAGTTAATAGAAAATTTTAGTTTCTATCAAATGTTTTCAAAACAATTGCTTAAAT
CAAGCTCATTAACTAAATAGGTTAATTTAGAAATTTATCTCAAAATTTAGAAATTATAGGGTTTAA
AATATAGTATGAGAAGTAGAGTACTGTAAGAATTTGTCCTGTTAAAATAAAAGGGGATTCAACTGG
TCGTGCTCCAATTCATGTTAATAAAATAATAATAATAACTATATATCAAAATAAGATTTGATTAAG
AGGATAAAATTGTAATCCTTGAGATTTATTTACATGTAAAATAGGAAGAATAAATAGGATTGCAAT
TGATATAACAAGTGCAATTACACCTCCTAATTTATTGGGGATAGAACGTAAAATAGCATAGGCAAA
TAAAAAATATCNNTNGNN

**> Sample 17**

Primer N1N-PDR

NGGNNNAATTGCACTTGTTATATCAATTGCAATCCTATTTATTCTTCCTATTTTACATGTAAATAAA
TCTCAAGGATTACAATTTTATCCTCTTAATCAAATCTTATTTTGATATATAGTTATTATTATTATTTT
ATTAACATGAATTGGAGCACGACCAGTTGAATCCCCTTTTATTTTAACAGGACAAATTCTTACAGTA
CTCTACTTCTCATACTATATTTTAAACCCTATAATTTCTAAATTTTGAGATAAATTTCTAAATTAACC
TATTTAGTTAATGAGCTTGATTTAAGCAATTGTTTTGAAAACATTTGATAGAAACTAAAATTTTCTA
TTAACTTTACTAATTTTAATTATTATAATAAAATAATTTTTAATCCAATAAAAAAAATAATATAACA
TAATGAAGCAGGTAAATAACTTTTTCAAACTAAATATATTAATTTATCATNNCGANNNC

Primer C3B-PDR

TGCTTCATTATGTTATATTATTTTTTTTATTGGATTAAAAATTATTTTATTATAATAATTAAAATTAG
TAAAGTTAATAGAAAATTTTAGTTTCTATCAAATGTTTTCAAAACAATTGCTTAAATCAAGCTCATT
AACTAAATAGGTTAATTTAGAAATTTATCTCAAAATTTAGAAATTATAGGGTTTAAAATATAGTAT
GAGAAGTAGAGTACTGTAAGAATTTGTCCTGTTAAAATAAAAGGGGATTCAACTGGTCGTGCTCCA
ATTCATGTTAATAAAATAATAATAATAACTATATATCAAAATAAGATTTGATTAAGAGGATAAAAT
TGTAATCCTTGAGATTTATTTACATGTAAAATAGGAAGAATAAATAGGATTGCAATTGATATAACA
AGTGCAATTACACCTCCTAATTTATTGGGGATAGAACGTAAAATAGCATAGGCAAATAAAAAATAT
CATTCNGNNN

**> Sample 18**

Primer N1N-PDR

NNNNNNTTAGGAGGTGTAATTGCACTTGTTATATCAATTGCAATCCTATTTATTCTTCCTATTTTAC
ATGTAAATAAATCTCAAGGATTACAATTTTATCCTCTTAATCAAATCTTATTTTGATATATAGTTATT
ATTATTATTTTATTAACATGAATTGGAGCACGACCAGTTGAATCCCCTTTTATTTTAACAGGACAAA
TTCTTACAGTACTCTACTTCTTATACTATATTTTAAACCCTATAATTTCTAAATTTTGAGATAAATTT
CTAAATTAACCTATTTAGTTAATGAGCTTGATTTAAGCAATTGTTTTGAAAACATTTGATAGAAACT
AAAATTTTCTATTAACTTTACTAATTTTAATTATTATAATAAAATAATTTTTAATCCAATAAAAAAA
ATAATATAACATAATGAAGCAGGTAAATAACTTTTTCAAACTAAATATATTAATTTATCATAACGA
ATTCGAGNNAANTA

Primer C3B-PDR

NTACCNTGCTTCATTATGTTATATTATTTTTTTTATTGGATTAAAAATTATTTTATTATAATAATTAA
AATTAGTAAAGTTAATAGAAAATTTTAGTTTCTATCAAATGTTTTCAAAACAATTGCTTAAATCAAG
CTCATTAACTAAATAGGTTAATTTAGAAATTTATCTCAAAATTTAGAAATTATAGGGTTTAAAATAT
AGTATAAGAAGTAGAGTACTGTAAGAATTTGTCCTGTTAAAATAAAAGGGGATTCAACTGGTCGTG
CTCCAATTCATGTTAATAAAATAATAATAATAACTATATATCAAAATAAGATTTGATTAAGAGGAT
AAAATTGTAATCCTTGAGATTTATTTACATGTAAAATAGGAAGAATAAATAGGATTGCAATTGATA
TAACAAGTGCAATTACACCTCCTAATTTATTGGGGATAGAACGTAAAATAGCATAGGCAAATAAAA
AATATCATTCNGN

**> Sample 19**

Primer N1N-PDR

NCCNATANNTTAGGAGGNGNNATTGCACTTGTTATATCAATTGCAATCCTATTTATTCTTCCTATTT
TACATGTAAATAAATCTCAAGGATTACAATTTTATCCTCTTAATCAAATCTTATTTTGATATATAGT
TATTATTATTATTTTATTAACATGAATTGGAGCACGACCAGTTGAATCCCCTTTTATTTTAACAGGA
CAAATTCTTACAGTACTCTACTTCTCATACTATATTTTAAACCCTATAATTTCTAAATTTTGAGATAA
ATTTCTAAATTAACCTATTTAGTTAATGAGCTTGATTTAAGCAATTGTTTTGAAAACATTTGATAGA
AACTAAAATTTTCTATTAACTTTACTAATTTTAATTATTATAATAAAATAATTTTTAATCCAATAAA
AAAAATAATATAACATAATGAAGCAGGTAAATAACTTTTTCAAACTAAATATATTAATTTATCATN
NNGANTTC

Primer C3B-PDR

GACTTNNTTATGTTATATTATTTTTTTTATTGGATTAAAAATTATTTTATTATAATAATTAAAATTAG
TAAAGTTAATAGAAAATTTTAGTTTCTATCAAATGTTTTCAAAACAATTGCTTAAATCAAGCTCATT
AACTAAATAGGTTAATTTAGAAATTTATCTCAAAATTTAGAAATTATAGGGTTTAAAATATAGTAT
GAGAAGTAGAGTACTGTAAGAATTTGTCCTGTTAAAATAAAAGGGGATTCAACTGGTCGTGCTCCA
ATTCATGTTAATAAAATAATAATAATAACTATATATCAAAATAAGATTTGATTAAGAGGATAAAAT
TGTAATCCTTGAGATTTATTTACATGTAAAATAGGAAGAATAAATAGGATTGCAATTGATATAACA
AGTGCAATTACACCTCCTAATTTATTGGGGATAGAACGTAAAATAGCATAGGCAAATAAAAAATAT
CATTCNGNN

**> Sample 23**

Primer N1N-PDR

NAATTGCANTTGTTATATCAATTGCAATCCTATTTATTCTTCCTATTTTACATGTAAATAAATCTCAA
GGATTACAATTTTATCCTCTTAATCAAATCTTATTTTGATATATAGTTATTATTATTATTTTATTAAC
ATGAATTGGAGCACGACCAGTTGAATCCCCTTTTATTTTAACAGGACAAATTCTTACAGTACTCTAC
TTCTCATACTATATTTTAAACCCTATAATTTCTAAATTTTGAGATAAATTTCTAAATTAACCTATTTA
GTTAATGAGCTTGATTTAAGCAATTGTTTTGAAAACATTTGATAGAAACTAAAATTTTCTATTAACT
TTACTAATTTTAATTATTATAATAAAATAATTTTTAATCCAATAAAAAAAATAATATAACATAATGA
AGCAGGTAAATAACTTTTTCAAACTAAATATATTAATTTATCATANNGANNNC

Primer C3B-PDR

ANNNNGCTTCATTATGTTATATTATTTTTTTTATTGGATTAAAAATTATTTTATTATAATAATTAAAA
TTAGTAAAGTTAATAGAAAATTTTAGTTTCTATCAAATGTTTTCAAAACAATTGCTTAAATCAAGCT
CATTAACTAAATAGGTTAATTTAGAAATTTATCTCAAAATTTAGAAATTATAGGGTTTAAAATATA
GTATGAGAAGTAGAGTACTGTAAGAATTTGTCCTGTTAAAATAAAAGGGGATTCAACTGGTCGTGC
TCCAATTCATGTTAATAAAATAATAATAATAACTATATATCAAAATAAGATTTGATTAAGAGGATA
AAATTGTAATCCTTGAGATTTATTTACATGTAAAATAGGAAGAATAAATAGGATTGCAATTGATAT
AACAAGTGCAATTACACCTCCTAATTTATTGGGGATAGAACGTAAAATAGCATAGGCAAATAAAA
AATATCATTCNGGNTN

**> Sample 856**

Primer N1N-PDR

TNATNNCACTTGTTATATCAATTGCAATCCTATTTATTCTTCCTATTTTACATGTAAATAAATCTCAA
GGATTACAATTTTATCCTCTTAATCAAATCTTATTTTGATATATAGTTATTATTATTATTTTATTAAC
ATGAATTGGAGCNNGACCAGTTGAATCCCCTTTTATTTTAACAGGACAAATTCTTACAGTACTCTAC
TTCTCATACTATATTTTAAACCCTATAATTTCTAAATTTTGAGATAAATTTCTAAATTAACCTATTTA
GTTAATGAGCTTGATTTAAGCAATTGTTTTGAAAACATTTGATAGAAACTAAAATTTTCTATTAACT
TTACTAATTTTAATTATTATAATAAAATAATTTTTAATCCAATAAAAAAAATAATATAACATAATGA
AGCAGGTAAATAACTTTTTCAAACTAAATATATNNATTTATCATNNNGAANN

Primer C3B-PDR

NTANCNTGCTTCATTATGTTATATTATTTTTTTTATTGGATTAAAAATTATTTTATTATAATAATTAA
AATTAGTAAAGTTAATAGAAAATTTTAGTTTCTATCAAATGTTTTCAAAACAATTGCTTAAATCAAG
CTCATTAACTAAATAGGTTAATTTAGAAATTTATCTCAAAATTTAGAAATTATAGGGTTTAAAATAT
AGTATGAGAAGTAGAGTACTGTAAGAATTTGTCCTGTTAAAATAAAAGGGGATTCAACTGGTCGTG
CTCCAATTCATGTTAATAAAATAATAATAATAACTATATATCAAAATAAGATTTGATTAAGAGGAT
AAAATTGTAATCCTTGAGATTTATTTACATGTAAAATAGGAAGAATAAATAGGATTGCAATTGATA
TAACAAGTGCAATTACACCTCCTAATTTATTGGGGATAGAACGTAAAATAGCATAGGCAAATAAAA
AATATCATTCTGGNN

**> Sample 872**

Primer N1N-PDR

TNTNATNNCACTTGTTATATCAATTGCAATCCTATTTATTCTTCCTATTTTACATGTAAATAAATCTC
AAGGATTACAATTTTATCCTCTTAATCAAATCTTATTTTGATATATAGTTATTATTATTATTTTATTA
ACATGAATTGGAGCACGACCAGTTGAATCCCCTTTTATTTTAACAGGACAAATTCTTACAGTACTCT
ACTTCTCATACTATATTTTAAACCCTATAATTTCTAAATTTTGAGATAAATTTCTAAATTAACCTATT
TAGTTAATGAGCTTGATTTAAGCAATTGTTTTGAAAACATTTGATAGAAACTAAAATTTTCTATTAA
CTTTACTAATTTTAATTATTATAATAAAATAATTTTTAATCCAATAAAAAAAATAATATAACATAAT
GAAGCAGGTAAATAACTTTTTCAAACTAAATATATTAATTTATCATNNNGAAANC

Primer C3B-PDR

NANTACCTGCTTCATTATGTTATATTATTTTTTTTATTGGATTAAAAATTATTTTATTATAATAATTA
AAATTAGTAAAGTTAATAGAAAATTTTAGTTTCTATCAAATGTTTTCAAAACAATTGCTTAAATCAA
GCTCATTAACTAAATAGGTTAATTTAGAAATTTATCTCAAAATTTAGAAATTATAGGGTTTAAAATA
TAGTATGAGAAGTAGAGTACTGTAAGAATTTGTCCTGTTAAAATAAAAGGGGATTCAACTGGTCGT
GCTCCAATTCATGTTAATAAAATAATAATAATAACTATATATCAAAATAAGATTTGATTAAGAGGA
TAAAATTGTAATCCTTGAGATTTATTTACATGTAAAATAGGAAGAATAAATAGGATTGCAATTGAT
ATAACAAGTGCAATTACACCTCCTAATTTATTGGGGATAGAACGTAAAATAGCATAGGCAAATAAA
AAATATCATTNNNNNN

**> Sample 874**

Primer N1N-PDR

NNAANTNNGGAGGNNNAATTGCNCTTGTTATATCAATTGCAATCCTATTTATTCTTCCTATTTTACA
TGTAAATAAATCTCAAGGATTACAATTTTATCCTCTTAATCAAATCTTATTTTGATATATAGTTATTA
TTATTATTTTATTAACATGAATTGGAGCACGACCAGTTGAATCCCCTTTTATTTTAACAGGACAAAT
TCTTACAGTACTCTACTTCTCATACTATATTTTAAACCCTATAATTTCTAAATTTTGAGATAAATTTC
TAAATTAACCTATTTAGTTAATGAGCTTGATTTAAGCAATTGTTTTGAAAACATTTTGATANNNA

Primer C3B-PDR

NNANTACCTGCTTCATTATGTGTATATTATTTTTTTTATTGGATTAAAAATTATTTTATTATAATAAT
TAAAATTAGTAAAGTTAATAGAAAATTTTAGTTTCTATCAAATGTTTTCAAAACAATTGCTTAAATC
AAGCTCATTAACTAAATAGGTTAATTTAGAAATTTATCTCAAAATTTAGAAATTATAGGGTTTAAA
ATATAGTATGAGAAGTAGAGTACTGTAAGAATTTGTCCTGTTAAAATAAAAGGGGATTCAACTGGT
CGTGCTCCAATTCATGTTAATAAAATAATAATAATAACTATATATCAAAATAAGATTTGATTAAGA
GGATAAAATTGTAATCCTTGAGATTTATTTACATGTAAAATAGGAAGAATAAATAGGATTGCAATT
GATATAACAAGTGCAATTACACCTCCTAATTTATTGGGGATAGAACGTAAAATAGCATAGGCAAAT
AAAAAATATCATTCNGNN
